# Prehospital transported patients: a resource for accessing prognostic risk factors

**DOI:** 10.1186/cc14587

**Published:** 2015-03-16

**Authors:** C Bech, M Brabrand, A Lassen

**Affiliations:** 1 Odense University Hospital, Odense, Denmark; 2 Sydvestjysk Sygehus Esbjerg, Denmark

## Introduction

The survival of patients transported by ambulance to the emergency department (ED) depends on clinical conditions, patient-related factors and organisational prehospital set up. Data and information concerning patients in the prehospital system could form a valuable resource for assessing potential risk factors associated with adverse outcomes and mortality. Our aim was to describe ambulance transports to the ED and identify prognostic factors accessible in the prehospital phase and associated with 7-day mortality.

## Methods

We included all adult patients (≥18 years) with a first-time ambulance transport to the ED at Odense University Hospital in the period 1 April 2012 to 30 September 2013. Ambulance personnel recorded vital signs and other clinical findings on a structured form on paper during the ambulance transport. Each contact was linked to information from population-based healthcare registers in order to identify comorbid conditions and information on mortality. Demographic factors and first registered vital sign were analysed by univariate logistic regression analysis, with 7-day mortality as outcome.

## Results

In total, 18,572 first-time ambulance contacts were identified in the period of inclusion. Overall 7-day mortality was 4.3% (95% CI = 4.0 to 4.6). Univariate analysis showed increasing age, Charlson Comorbidity Index ≥2, vital parameters outside the normal reference range and summoned physician-assisted mobile emergency care units to be associated with 7-day mortality. Further analyses are currently being carried out.

## Conclusion

We found that several prehospital-registered vital signs recorded by ambulance personnel at first contact with the patient were prognostic factors of 7-day mortality.

